# Efficacy and Toxicity Assessment of Different Antibody Based Antiangiogenic Drugs by Computational Docking Method

**DOI:** 10.1155/2016/7053712

**Published:** 2016-03-07

**Authors:** Sayan Mukherjee, Gopa Chatterjee, Moumita Ghosh, Bishwajit Das, Durjoy Majumder

**Affiliations:** ^1^Department of Physiology, West Bengal State University, Berunanpukuria, Malikapur, Barasat, Kolkata 700 126, India; ^2^Society for Systems Biology & Translational Research, No. 103, Block C, Bangur Avenue, Kolkata 700 055, India

## Abstract

Bevacizumab and trastuzumab are two antibody based antiangiogenic drugs that are in clinical practice for the treatment of different cancers. Presently applications of these drugs are based on the empirical choice of clinical experts that follow towards population based clinical trials and, hence, their molecular efficacies in terms of quantitative estimates are not being explored. Moreover, different clinical trials with these drugs showed different toxicity symptoms in patients. Here, using molecular docking study, we made an attempt to reveal the molecular rationale regarding their efficacy and off-target toxicity. Though our study reinforces their antiangiogenic potentiality and, among the two, trastuzumab has much higher efficacy; however, this study also reveals that compared to bevacizumab, trastuzumab has higher toxicity effect, specially on the cardiovascular system. This study also reveals the molecular rationale of ocular dysfunction by antiangiogenic drugs. The molecular rationale of toxicity as revealed in this study may help in the judicious choice as well as therapeutic scheduling of these drugs in different cancers.

## 1. Introduction

Solid tumor survives by the process of angiogenesis. Angiogenesis is a physiological process by which microvessels are developed around the tumor mass. Tumor cells secrete a variety of tumor associated growth factors (TAF) like VEGF (vascular endothelial growth factor), TGF (transforming growth factor), EGF (epidermal growth factor), PDGF (platelet-derived growth factor), PAI, and TSP-1 to promote the process of angiogenesis [1, 2]. The attempt to sequester these factors is known as antiangiogenic (AAG) therapy. It is suggested that AAG therapy can supplement ongoing chemotherapy, for example, with docetaxel, platinum based therapies, paclitaxel, vinorelbine, and gemcitabine especially when they have failed or are not tolerated [3]. For several types of cancer, application of different AAG drugs in combination with the conventional MTD (maximum tolerable dosing strategy) chemotherapy has shown positive results with reduced (chemotherapy related) toxicities [4].

In control of tumor growth, anti-VEGF antibody is being developed. Application of Avastin (bevacizumab), the commercially available anti-VEGF, has a remarkable success in the control of tumor growth in different clinical trials. For AAG therapy another antibody molecule known as trastuzumab has developed. Trastuzumab has the ability to inhibit a variety of other angiogenic molecules, namely, transforming growth factor (TGF), Ang-1, PAI-1, and thrombospondin 1 (TSP1) that might also respond to HER signaling [5].

Bevacizumab was the first antiangiogenic drug that was approved by the U.S. Food and Drug Administration (FDA) in 2004 for different metastatic cancers either alone or in combination with standard chemotherapy (Table 1). It is a recombinant humanized monoclonal IgG1 antibody that binds to and inhibits the biologic activity of human vascular endothelial growth factor (VEGF). Bevacizumab contains human framework regions and the complementarity-determining regions of a murine antibody that binds to VEGF. Bevacizumab is produced in a Chinese Hamster Ovary mammalian cell expression system. Interaction of bevacizumab with VEGF prevents the interaction of VEGF to its receptors (Flt-1 fms-like tyrosine kinase-1—VEGFR1 receptor and KDR kinase insert domain containing receptor—VEGFR2 receptor) on the surface of endothelial cells. This prevents blood vessel proliferation and in response retardation of metastatic tumor growth occurs [6]. However, application of bevacizumab in patients produces several side effects which include arterial thromboembolic events, hypertension, proteinuria, reversible leukoencephalopathy syndrome, skin rash, pulmonary hypertension, mesenteric venous occlusion, gastrointestinal ulcer and perforation, intestinal necrosis, gallbladder perforation, anastomotic ulceration, pancytopenia, necrotizing fasciitis, wound healing complications, osteonecrosis of the jaw, renal thrombotic microangiopathy (manifested as severe proteinuria), PRES (posterior reversible encephalopathy syndrome), hemorrhage, body as a whole polyserositis, and different forms of eye disorders such as increase in intraocular pressure with inflammation; retinal detachment; conjunctival, vitreous, or retinal hemorrhage; vitreous floaters; ocular hyperemia, pain, or discomfort. However, histopathological kidney lesion like glomerular lesion and interstitial nephritis occur less frequently. Anti-VEGF mediated hypertension is not well understood; nitric oxide pathway inhibition, rarefaction, and oxidative stress may be responsible for such pathogenesis [5, 7–10].

Trastuzumab is a IgG1 kappa monoclonal humanized antibody, produced in CHO cell line, approved by FDA in 2006 for treatment regimen containing doxorubicin, cyclophosphamide, and paclitaxel for the adjuvant treatment of women with node-positive, Her-2 overexpressing breast cancer. It is active against the human epidermal growth factor receptor 2 or Her-2/Neu and the binding of trastuzumab leads to complement mediated killing of the HER-2 positive cells (Table 1) [11, 12]. Though there is evidence of a significant prolongation in disease-free survival in women receiving trastuzumab and chemotherapy compared to those receiving chemotherapy alone, it produces four types of toxicities which need special clinical attention [13].

*(1) Cardiomyopathy*. There is a 4- to 6-fold increase in the incidence of symptomatic left ventricular myocardial dysfunction (decline in left ventricular ejection fraction) among patients receiving Herceptin as a single agent or in combination therapy compared with those not receiving Herceptin. Moreover, it causes cardiac arrhythmias, hypertension, disabling cardiac failure, cardiomyopathy, and cardiac death.

*(2) Infusion Reactions*. Serious and fatal infusion reactions have been reported. Infusion reactions consist of a symptom complex characterized by fever and chills and on occasion included nausea, vomiting, pain (in some cases at tumor sites), headache, dizziness, dyspnea, hypotension, rash, and asthenia. Severe reactions including bronchospasm, anaphylaxis, angioedema, hypoxia, and severe hypotension were usually reported during or immediately following the initial infusion.

*(3) Chemotherapy Induced Neutropenia*. In randomized, controlled clinical trials the per-patient incidences of NCI CTC Grade 3 to 4 neutropenia and of febrile neutropenia were higher in patients receiving Herceptin in combination with myelosuppressive chemotherapy as compared to those who received chemotherapy alone.

*(4) Pulmonary Toxicity*. Herceptin use can result in serious and fatal pulmonary toxicity. Pulmonary toxicity includes dyspnea, interstitial pneumonitis, pulmonary infiltrates, pleural effusions, noncardiogenic pulmonary edema, pulmonary insufficiency and hypoxia, acute respiratory distress syndrome, and pulmonary fibrosis. Such events can occur as sequelae of infusion reactions [14].

Though both drugs are being in clinical use as antiangiogenic drugs, their pharmacological evaluation specially the efficacy and/or toxicity assessment in quantitative terms has not been evaluated. Previously, the molecular rationale of off-target toxicity of different drugs is established by using molecular docking interaction method between an array of receptors present within the physiological system and different adjuvant drugs of breast cancer [15]. Using the same approach, pharmacological assessment of different arsenic chelator drugs is evaluated [16]. The present work investigates the molecular portrayals in quantitative terms on the efficacy as well as the above-mentioned side effects caused by different antibody based antiangiogenic drugs.

## 2. Materials and Methods

### 2.1. Software and Database

For the present work we have used Hex, an open source, freely available software for academic use. The present work is done with the data resources that are available in the public domain.  Table 2 provides the source of data availability used in this study. The Protein Data Bank (PDB) (http://www.rcsb.org/) is a worldwide repository for the processing and distribution of 3D biological macromolecular structure data [17]. Protein structures may be downloaded from the site with specific keywords or a PDB alphanumeric filename. The Drug Bank (http://redpoll.pharmacy.ualberta.ca/drugbank/) database [18] is a unique bioinformatics and cheminformatics resource that combines detailed drug (i.e., chemical, pharmacological, and pharmaceutical) data with comprehensive drug target (i.e., sequence, structure, and pathway) information. The database contains nearly 4300 drug entries including >1000 FDA-approved small molecule drugs, 113 FDA-approved biotech (protein/peptide) drugs, and >3000 experimental drugs. Additionally, more than 6000 protein (i.e., drug target) sequences are linked to these drug entries. Each Drug Card entry contains more than 80 data fields with half of the information being devoted to drug/chemical data and the other half devoted to drug target or protein data.

### 2.2. Molecular Docking

Molecular docking is performed using Hex program. Molecular docking helps in predicting the intermolecular interactions after forming an intermolecular complex between two constituent molecules. It uses spherical polar Fourier (SPF) correlations to accelerate the calculations. Using this program we have docked between receptor/enzyme and AAG drug (antibody)/drug in different combinations [19, 20]. Briefly the steps are as follows:

(1) From the File menu the Receptor and Ligand files were opened.
(2) When two molecules are loaded the scene origin is taken as the midpoint between the two molecular centroids. Although both receptor and ligand move during docking, generally more motion is assigned to the ligand and keeping the receptor fixed though this can be changed by pressing the Select origin button.
(3) There are few Protein-Protein software programs that give output in terms of energy; Hex is an exception although the energy output is closer to internal energy (Δ*U*). The energy is a function of the distance range chosen by the two origins and also the angles between them. So *E* = *f*(*x*, *y*, *z*) and *E* = *f*(*φ*, *ψ*, *θ*).
(4) Now the relation between Δ*G* and Δ*U* can be obtained from thermodynamics. We know entropy *S*, enthalpy *H*, and Gibb's free energy Δ*G* = Δ*H* − *T*Δ*S*. So at constant pressure, Δ*G* = Δ*U* + *P*Δ*V* − *T*Δ*S*. Hex considers only *E*_total_Δ*U* (total internal energy) and tends to neglect the entropy term that is the hydrophobic interactions; however, this corresponds as error at 100's of (+ or −) KJ/mol compared to experimental condition [21].
(5) After the Docking is activated from the Controls menu, the Energy is output together with the diagram of the docked complex.

### 2.3. Visualization of Drug-Protein and Protein-Protein Complexes

For viewing the results of hex docking, we used Hex itself. After docking, the distance between two different molecules is also determined.

## 3. Results

Rigorous docking experiments had been performed to assess the comparative efficacy between two clinically used AAG drugs and their cross-reactivity to different receptors and/or enzymes within the physiological system. The AAG drugs, the receptors, and the corresponding docking results are listed in Table 3.

**Figure 1:**
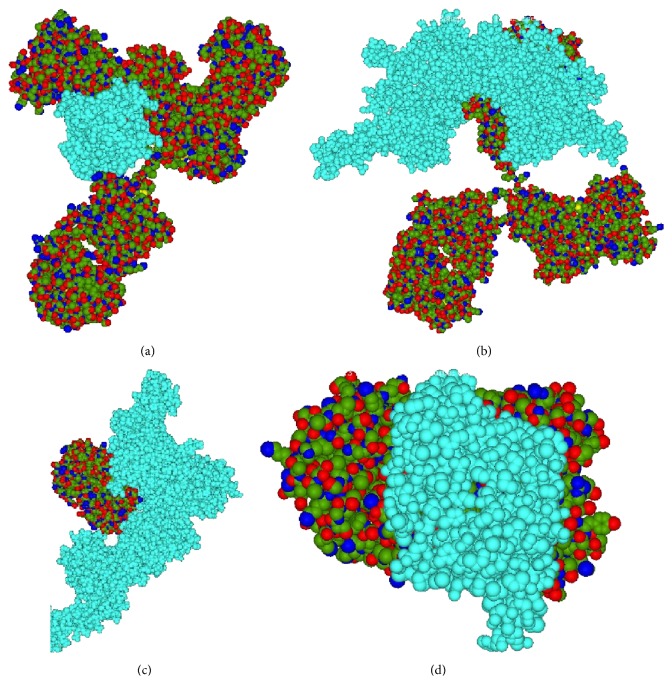
Docked conformation of bevacizumab to VEGF (a) and Her-2/Neu receptor (b), and trastuzumab to Her-2/Neu (c) and VEGF receptor (d); the receptor protein is in cyan color.

Our docking study reveals that the binding affinity of bevacizumab to VEGF receptor (−741.08) is much higher than its binding to Her-2/Neu receptor (−376.03). Binding affinity of trastuzumab to VEGF receptor (−721.08) is higher than Her-2/Neu receptor (−562.51) though it is much higher than the binding affinity of bevacizumab to Her-2/Neu receptor (−376.03) (Table 3) (Figure 1).

**Figure 2:**
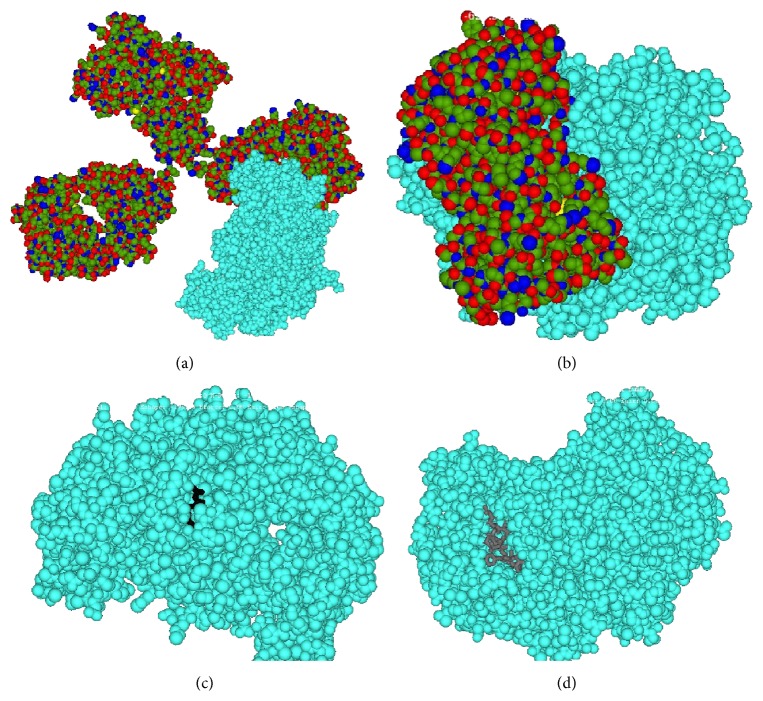
Docked conformation of bevacizumab (a), trastuzumab (b), NNA (c), and substance P (d) to NO synthase (in cyan color).

Both AAG drugs bind to beta-2, tyrosine kinase (TK) receptor, and NO synthase which indicate their antiangiogenic potentiality. Interestingly, compared to bevacizumab, trastuzumab has the higher binding affinity to beta-1, beta-2, tyrosine kinase receptors, and NO synthase. This result may indicate that antiangiogenic potentiality of trastuzumab may be higher than bevacizumab. However, these results also indicate the reason of hypertension and cardiac myopathy in trastuzumab treated patients and why they need special attention when they show symptoms of hypertension and cardiac myopathy. Interestingly, the majority of cases, binding affinity of both the AAG drugs to different receptors/enzymes, are much higher compared to the known ligands (antagonist/agonist) to those receptors/enzymes. Trastuzumab has the highest binding towards NO synthase (−743.53) among the studied receptor/enzyme molecules in this work. Contrarily, bevacizumab has much less binding affinity towards NO synthase compared to their known ligands (antagonist/agonist); therefore there is much less chance of cardiac myopathy by this drug (Table 3) (Figure 2). Moreover, this data also indicates that, in bevacizumab treated patients, synergistic application of immunotherapy is also possible.

Bevacizumab does not have any binding affinity to angiotensin II type I (AT 1) and beta-1 receptor. However, binding affinity of bevacizumab is very close to angiotensin converting enzyme (ACE) antagonist lisinopril. Compared to trastuzumab it has also less affinity for ACE; so the cause of observed hypertension by the use of bevacizumab may be due to destruction of microvessels or it may act as an agonist for ACE, whereas higher binding of trastuzumab to ACE may indicate the cause of infusion reaction by trastuzumab [5].

**Figure 3:**
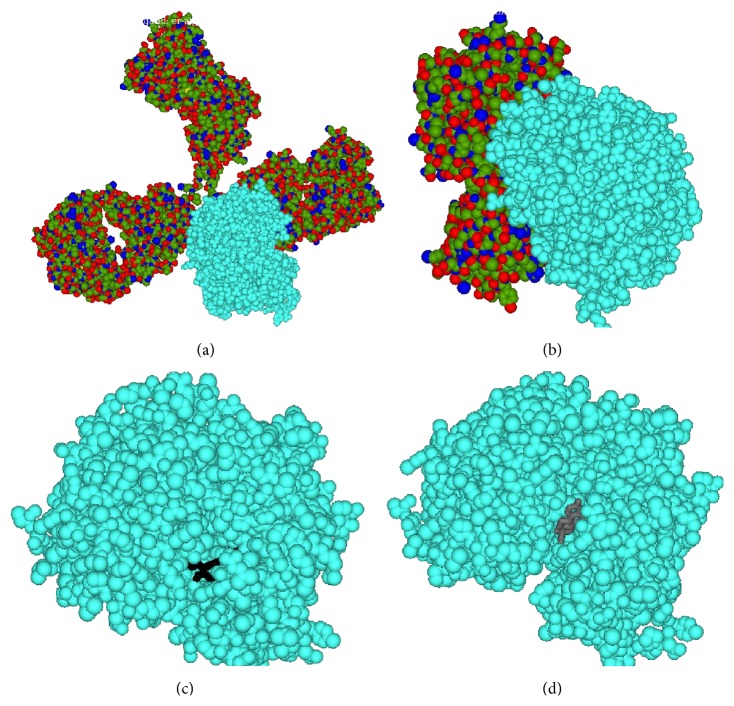
Docked conformation of bevacizumab (a), trastuzumab (b), tamoxifen (c), and ethinyl estradiol (d) to estrogen receptor alpha (in cyan color).

Both AAG drugs showed much higher binding affinity towards estrogen receptor (ER); hence, both of them would be effective in estrogen positive breast/ovarian cancer. Compared to bevacizumab, trastuzumab has increased binding affinity to ER-alpha; therefore trastuzumab may also be more effective in controlling of estrogen positive breast/ovarian cancer (Table 3) (Figure 3). The docked conformation of two AAG drugs to the androgen receptor (AR) showed that the binding affinity of two AAG drugs has much higher binding affinity to it compared to its known ligands. These results indicate that AAG drugs are effective in other cancers related to steroidal hormones. For this type of receptor trastuzumab also showed higher binding affinity than bevacizumab.

**Figure 4:**
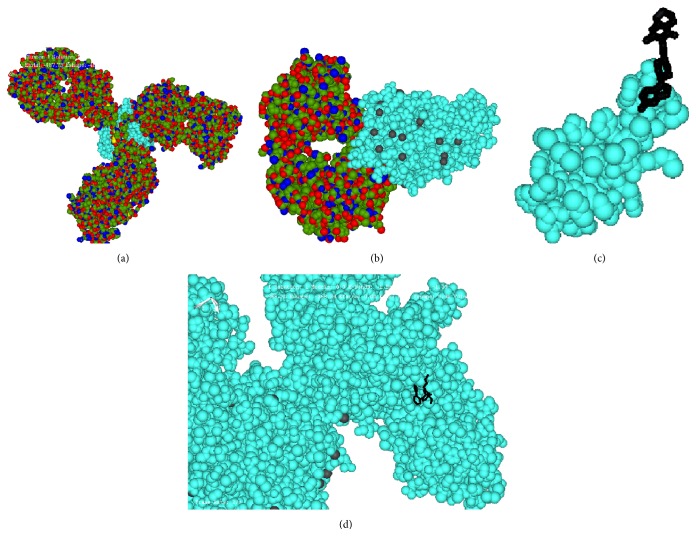
Docked conformation of bevacizumab (a), trastuzumab (b), risperidone (c), and cabergoline (d) to dopamine-2 receptor (in cyan color).

Our docking results also reveal that both AAG drugs have a higher binding affinity towards dopamine-2 receptor [bevacizumab (−513.8) > trastuzumab (−299.85)]; this data indicates why the symptom of nausea is more pronounced with bevacizumab treatment than with trastuzumab (Figure 4). Both the AAG drugs have almost equal binding affinity to histamin-2 receptor and both have much higher binding affinity with respect to known ligands (antagonist and agonist). Similar trend is also seen for both types of GABA receptors with lesser for bevaczumab to GABA-A (Table 3). Simulation data also suggest that both AAG drugs have very high binding (compared to its known ligands) towards adrenoceptor alpha 2a receptor (ADRA2A), which is very important receptor for retinal functioning. So this study also reveals the molecular rationale of ocular dysfunction by AAG drugs.

## 4. Discussion

Long-term treatment is necessary for different cancers. But conventional chemotherapies have toxic side effect. Hence there is a search for specificity in cancer treatment. It is expected that monoclonal antibody based drugs may provide that way out. Though both bevacizumab and trastuzumab are FDA-approved antiangiogenic drugs in clinical use for the therapy of a wide variety of cancers, their efficacy and/or selection for clinical use are in empirical state. There are scanty report regarding their selection and drug scheduling for different cancers. Different clinical trials suggest for combination therapy even with the conventional chemotherapy. So our computational prediction is useful due to scanty data availability.

Though different analytical and simulation based studies showed that the efficacies of such drugs may have better in control of tumor growth, different clinical reports suggest that these monoclonal antibody based drugs do not qualify towards the expected criteria of overcoming the physiological toxicity [22, 23]. This, in turn, may limit the wider acceptance of AAG drugs for cancer treatment. The issue of drug related toxicity has become the focal theme with the rise of systems biology and for a decade long this issue has been addressed in the area of cancer systems biology which reinforces the need of theoretical as well as simulation study [24–30].

Previously different docking studies (using Autodock and/or Hex) have proved the efficacy of macromolecular interactions as well as drug-macromolecular interactions, where docking results indicate the fact that the more the negative energy, the more the binding efficacy [15, 16, 19–21]. Previous work also suggests that off-target toxicity can be studied through docking based method and, for this, docked result between a drug and its off-target is compared with the docked result between the same target and its known ligand/drug. If the two results are similar or near to similar then off-target binding can be concluded. If former result is greater than or near the value of the latter then off-target toxicity is possible. Again comparison of binding data of drug with off-target, target with known antagonist/agonist, and the clinical findings/reports may hint towards the molecular rationale of pharmacological/toxicological mechanisms [15].

The present docking based study indicates that trastuzumab has better antiangiogenic potentiality compared to bevacizumab; however, it has much more side effects on the cardiovascular system. The major problems of using AAG drugs are nausea, hypertension, gastric ulceration, and ocular damage. Our docking study reveals its molecular rationale as the binding affinity of both AAG drugs on D2, beta-2, GABA, ACE, Ca-channel, H2, and ADRA2A; most importantly trastuzumab has the highest binding affinity for NOS; this may be the probable cause that in trastuzumab treatment cardiomyopathy needs special attention. Our data also indicate that, in bevacizumab treated patients, synergistic application of other immunotherapy is also possible.

This in silico study may provide the molecular rationale of toxicity within the physiological system and hints towards the probable cause of pathophysiological alteration with the application of AAG drugs in patients. With the availability of time dependent data related to the onset toxicological symptoms by AAG drugs, a correlation study between the two would then be beneficial in the future particularly for the calibration of drug scheduling. Hence, generation of dynamical database regarding cancer treatment along with the onset of toxicological symptoms in humans (for different drugs) is needed. Towards this aspect this study has the significance.

## Figures and Tables

**Table 1:** FDA-approved different antibody based antiangiogenic drugs.

Drug (MW)	Drug target	Recommended dose (half-life)	Types of cancer that are recommended for treatment
Bevacizumab, available as Avastin (149 KD)	VEGF receptor	IV infusion of 5–10 mg/kg body wt. in every 2 or 3 weeks (~20 days)	Metastatic colon, colorectal, cervical and peritoneal cancer with std. CT; platinum rst. ovarian or fallopian tube cancer; metastatic HER2 negative breast cancer; renal carcinoma; first-line treatment of non-small-cell lung cancer; second-line treatment of glioblastoma, different types of hematological malignancies

Trastuzumab, available as Herceptin (145.5 KD)	HER-2	IV infusion of 2–8 mg/kg body wt. in every week (~28.5 days)	HER-2 overexpressing including ER/PR negative and node positive or negative breast cancer in combination with either anthracycline based (paclitaxel or docetaxel) or cisplatin and capecitabine or 5-fluorouracil or carboplatin chemotherapy; HER-2 positive metastatic gastric or gastroesophageal junction endocarcinoma without prior chemotherapy

IV: intravenous administration; std.: standard; CT: chemotherapy; rst.: resistant; TK: tyrosine kinase; wt.: weight.

**Table 2:** Sources of PDB files used in the study.

Receptor/drug	Code/accession number	Website reference
Androgen receptor	PDB ID 2YHD (edited)	http://www.rcsb.org/pdb/home/
Beta-1 receptor	PDB ID 2VT4 (edited)	”
Beta-2 receptor	PDB ID 2R4R (edited)	”
Dopamine-2 (D2) receptor	PDB ID 2YOU (edited)	”
Estrogen-*α* (ER-*α*) receptor	PDB ID 1X7E (edited)	”
GABA-A receptor	PDB ID 3D32 (edited)	”
GABA-B receptor	PDB ID 1SRZ (edited)	”
Histamine (H2) receptor	Univ. of Michigan	http://www.personal.umich.edu/~him/H2A.htm ^*∗*^
Angiotensin II type 1 (AT 1) receptor	PDB ID 3D0G (edited)	http://www.rcsb.org/pdb/home/
Nitric oxide synthase (NOS)	PDB ID 1ED5 (edited)	”
Tyrosine kinase (TK)	PDB ID 1M17 (edited TK domain of EGF)	http://www.rcsb.org/pdb/home/
Adrenoceptor alpha 2a (ADRA2A)	PDB ID 1HLL	”
Angiotensin converting enzyme (ACE)	PDB ID 4UFA	”
Ca-channel	PDB ID 1T3L	”
Her-2/Neu	PDB ID 1S78 (edited)	http://www.rcsb.org/pdb/home/
VEGF (vascular endothelial growth factor)-C	PDB ID 2XIX (edited)	”
Angiotensin II	PDB ID 1ZV0 (edited)	”
NNA (N-nitro-L-arginine)	PDB ID 8NSE	”
Imatinib	Primary Acc. number DB00619	http://www.drugbank.ca
Cyproterone	Primary Acc. number DB04839	”
Nandrolone	Primary Acc. number DB00984	”
Propranolol	Primary Acc. number DB00571	”
Epinephrine	Primary Acc. number DB00668	”
Risperidone	Primary Acc. number DB00734	”
Cabergoline	Primary Acc. number DB00248	”
Flumazenil	Primary Acc. number DB01205	”
Diazepam	Primary Acc. number DB00829	”
Tamoxifen	Primary Acc. number DB00675	http://www.drugbank.ca
Ethinyl estradiol	Primary Acc. number DB00977	”
Saclofen	Marvin sketched at http://www.chemaxon.com and saved as pdb	http://www.chemaxon.com
Baclofen	Primary Acc. number DB00181	http://www.drugbank.ca
Ranitidine	Primary Acc. number DB00863	”
Betazole	Primary Acc. number DB00272	”
Losartan	Primary Acc. number DB00678	”
Yohimbine	Primary Acc. number DB01392	”
Clonidine	Primary Acc. number DB00575	”
Lisinopril	Primary Acc. number DB00722	”
Nifedipine	Primary Acc. number DB01115	”
Bevacizumab/Avastin	Primary Acc. number DB00112	”
Trastuzumab/Herceptin	Primary Acc. number DB00072	”
Substance P	Primary Acc. number DB05875	”

^*∗*^accessed on 4-Dec-2011.

**Table 3:** Hex performed docked energies of drug (antibody) − receptor/enzyme and drug (antagonist/agonist) − receptor/enzyme in *E*_total_Δ*U* (KJ/mol). Data in parentheses indicate intermolecular interacting site and distance.

Receptor/enzyme	Drug/ligand			
	Bevacizumab/Avastin	Trastuzumab/Herceptin	Known antagonist	Known agonist
(1) Androgen	−217.19 [LYS17, A, 2HZ (Avastin) − ASP282, B, OD2 (androgen) = 5.01 Å]	−680.07 [HIS689, A, CE1 (Herceptin) − ASN681, A, OD1 (androgen) = 5.44 Å]	Cyproterone = −195.13	Nandrolone = −239.81

(2) Beta-1	0.00 [D10406, D, C2 (Avastin) − SER215, C, OG (beta-1) = 7.60 Å]	−480.80 [SOG404, D, O2 (Herceptin) − ARG157, C, HE (beta-1) = 3.33 Å]	Propranolol = −205.44	Epinephrine = −150.35

(3) Beta-2	−200.73 [VAL169, H, CG1 (Avastin) − LEU160, L, CD2 (beta-2) = 3.96 Å]	−655.19 [ASN57, H, OD1 (Herceptin) − LYS270, A, 1HZ (beta-2) = 3.54 Å]	Propranolol = −218.25	Epinephrine = −148.88

(4) Dopamine-2 (D2)	−513.80 [ARG13, C, 1HH1 (Herceptin) − ASN343, B, CB (D2) = 7.69 Å]	−299.85 [ARG13, C, N (Herceptin) − TYR87, A, CZ (D2) = 6.13 Å]	Risperidone = −114.72	Cabergoline = −130.19

(5) Estrogen-*α* (ER-*α*)	−327.20 [GLU542, B, CB (Avastin) − HIS687, D, CB (estrogen-*α*) = 5.76 Å]	−712.68 [GLN695, D1HE2 (Herceptin) − LEU372, B, CD2 (ER-*α*) = 5.75 Å]	Tamoxifen = −223.14	Ethinyl estrogen = −186.48

(6) GABA-A	−432.29 [GLU75, A, OE2 (Avastin) − TYR27, B, HH (GABA-A) = 7.46 Å]	−680.98 [GLU81, A, CB (Herceptin) − LYS68, B, CG (GABA-A) = 2.87 Å]	Flumazenil = −69.88	Diazepam = −127.70

(7) GABA-B	−626.88 [THR23, D, CA (Avastin) − PHE112, A, HE2 (GABA-B) = 2.02 Å]	−641.84 [THR267, B, HG1 (Herceptin) − THR114, A, HG2 (GABA-B) = 1.14 Å]	Saclofen = −170.79	Baclofen = −176.46

(8) Histamine-2 (H2)	−677.13 [LYS241, B, CB (Avastin) − VAL51, A, CG1 (H2R) = 5.99 Å]	−687.69 [VAL176, B, CB (Herceptin) − ILE77, A, CD1 (H2R) = 5.78 Å]	Ranitidine = −224.76	Betazole = −144.17

(9) Angiotensin II type 1 (AT1)	0.00 [NAG91, F, 04 (Avastin) − GLN287, B, CB (AT1) = 4.97 Å]	−369.66 [THR486, F, CB (Herceptin) − TYR484, B, CD2 (AT1) = 4.34 Å]	Losartan = −268.32	Angiotensin II = −460.09

(10) NO synthase (NOS)	−69.39 [THR392, A, HG1 (NOS) − ALA424, B, CB (NOS) = 3.04 Å]	−745.53 [ASN206, B, 2HD2 (Herceptin) − ARG257, B, 2HH1 (NOS) = 5.19 Å]	NNA = −196.39	Substance P = −458.63

(11) Tyrosine kinase (TK)	−294.73 [GLN287, B, CB (Avastin) − ALA726, A, CB (TK) = 3.04 Å]	−567.61 [ASP17, A, OD1 (Herceptin) − PRO675, A, CB (TK) = 3.69 Å]	Imatinib = −283.36	Not known

(12) Adrenoceptor alpha 2a receptor (ADRA2A)	−633.40 [ALA100, A, CB (Avastin) − GLN81, B, OE1 (alpha 2a) = 4.94 Å]	−740.85 [TRP440, D, CE3 (Herceptin) – GLN341, C, OE1 (alpha 2a) = 7.80 Å]	Yohimbine = −182.45	Clonidine = −132.03

(13) Angiotensin converting enzyme (ACE)	−243.12 [LYS363, A, 3HZ (Avastin) − LYS414, A, HZ2 (ACE) = 0.29 Å]	−403.71 [SER210, A, HG (Herceptin) − THR212, B, HG1 (ACE) = 2.74 Å]	Lisinopril = −234.14	Not known

(14) Ca-channel	−241.56 [LEU434, F, CD2 (Avastin) − ARG302, B, NH1 = 5.43 Å]	−652.58 [GLU431, F, 0E2 (Herceptin) − ARG302, B, CG = 5.34 Å]	Nifedipine = −221.68	Not known

(15) Her-2/Neu	−376.03 [THR231, B, CGZ (Avastin) − GLU18, C, OE2 (Her-2) = 10.36 Å]	−562.51 [LYS213, B, 3HZ (Herceptin) − ARG12, C, HE (Her-2) = 2.18 Å]	Not known	Not known

(16) VEGF	−741.08 [GLN79, V, OE1 (Avastin) − LYS16, W, CB (VEGF) = 10.92 Å]	−721.08 [ALA13, A, CB (Herceptin) − GLN37, W, OE1 (VEGF) = 1.13 Å]	Not known	Not known
